# In Vitro Degradation of Chlorpyrifos by the Ruminal Microbes: Insights from the Rumen Metagenome

**DOI:** 10.3390/microorganisms14030581

**Published:** 2026-03-04

**Authors:** Pradeep Kumar Malik, Archit Mohapatra, Shraddha Trivedi, Atul Purushottam Kolte, Artabandhu Sahoo, Raghavendra Bhatta

**Affiliations:** 1 ICAR-National Institute of Animal Nutrition and Physiology, Bengaluru 560030, India; pradeep.malik@icar.org.in (P.K.M.); arch10moha@gmail.com (A.M.); shraddha.trivedi_8@yahoo.com (S.T.); directornianp@gmail.com (A.S.); 2 Indian Council of Agricultural Research, New Delhi 110001, India; ragha0209@yahoo.com

**Keywords:** chlorpyrifos, degradation, metagenome, microbial diversity, rumen

## Abstract

In vitro studies were conducted in a series to investigate if the ruminal microbes are capable of degrading chlorpyrifos. This in vitro study presents the results from three experiments: Exp. I was conducted without feed, while Exp II and III were conducted with feed, either with or without methanol for dissolving chlorpyrifos, respectively. A basal diet comprising finger millet straw and concentrate was prepared. Incubation medium with feed but without chlorpyrifos served as the control. A total of six replicates each of control and chlorpyrifos spiked were used for the incubation. The pesticide concentration in the incubation medium before and after 24 h of incubation was analyzed using GC-MS/MS. The genomic DNA was isolated from the incubation fluid of the individual samples, and the shotgun metagenomic sequencing was performed. The clean reads were taxonomically classified using the Kraken2 database, and microbial classification at different taxonomic ranks was separated using Pavian v1.0. The microbial genes in the metagenome data were predicted and assigned functional roles using the MetaErg v1.2.3 pipeline. The assigned KEGG Orthology (KO), EC numbers (Enzyme Commission number), Gene Ontology (GO), and corresponding NCBI taxonomy information relevant to chlorpyrifos metabolism/degradation were retrieved. Results from the study revealed that the chlorpyrifos concentration was decreased from 5.78 to 1.64 ppm over 24 h of in vitro incubation with feed. Similar alpha and beta diversity indices between control and chlorpyrifos treatments revealed that the richness and the evenness of the microbial population were not affected by the presence of chlorpyrifos in the rumen fluid. There was no difference in the microbiota affiliated to the major phyla such as Bacteroidota, Fibrobacterota, Bacillota, and Pseudomonadota. The EC 3.1.8.1, EC 3.1.3.1, EC 1.14.13.-, and EC 1.1.1.- reported for chlorpyrifos degradation were not detected in the metagenome, and only EC 3.1.1.1 was identified, which demonstrated that degradation of chlorpyrifos was carried out by the affiliated enzyme carboxylesterase. The presence of GO:0004035, GO:0004364, GO:0019637, GO:0016791, and GO:0042178 in the metagenome strengthens that the chlorpyrifos degradation in the present study was primarily assigned to the rumen microbiota. This in vitro study provided insights into the rumen microbiota involved in the chlorpyrifos degradation and the initial clue that the rumen microbes are capable of degrading chlorpyrifos. Further, the animal studies in different species with the variable levels of chlorpyrifos are also warranted to confirm the efficacy of rumen microbes in mixed syntrophy and determine the threshold capabilities of the ruminal microbes.

## 1. Introduction

Worldwide, 3.70 million tons of pesticides in terms of active ingredients are used in agriculture [1]. Chlorpyrifos is an organophosphate pesticide extensively used in a wide variety of food and feed crops to control insects and pests [2]. Recently, during the Stockholm Convention in Geneva to eliminate hazardous chlorpyrifos, many countries agreed to a complete ban on the use of chlorpyrifos pesticide; however, some of the countries sought exemptions for its use in selected crops for another five years [3]. In India, the Ministry of Agriculture and Farmers Welfare recently banned the usage of 46 pesticides, while nine pesticides have been kept under a watchlist with restricted use [4]. The pesticide residue could transfer from the agricultural crop to the animal feeds that are mostly obtained as by-products from the main agricultural crops [5]. The pesticide is absorbed and accumulated in the vegetative parts of the plants, such as leaves and stalks, which are used as feed for the livestock. Chlorpyrifos, despite the low aqueous solubility, highly volatile nature, and moderate persistence in soil, could transfer to the stalks, leaves, and grains at a lower concentration than the soil [6].

There are many reports on the biodegradation of chlorpyrifos in the soil [7,8,9,10]. Microbial degradation in the soil is one of the prominent mechanisms for chlorpyrifos degradation carried out by the microbial enzymes [11]. *Pseudomonas*, *Bacillus*, *Enterobacter*, *Stenotrophomonas*, *Sphingomonas,* and *Verticillium* are the prominent genera of microbes involved in the biodegradation of chlorpyrifos in the soil. Chlorpyrifos degradation by the microorganisms has been reported during the silage making over 90 days with a lesser efficiency than the degradation of beta-cypermethrin and tebuconazole [12]. Similarly, the degradation of chlorpyrifos by lactic acid bacteria during the ensiling process is also reported by Wang et al. [13]. These reports suggest that chlorpyrifos can be degraded by the soil microorganisms; however, the soil ecosystem does not resemble the rumen ecosystem, as the former is an external, aerobic, open environment driven by sunlight and rainfall, while the latter is an internal, strictly anaerobic, closed environment and host animal-dependent system. These two distinct ecosystems possess a diverse type of microbiota adapted to the specialized environment and carry different functions [14]. Similarly, the ensiling process and rumen ecosystem are also not the same, as the ensiling fermentation takes place in a silo, whereas the fermentation of ingested feed occurs in the rumen inside the animal’s body. Further, there is a distinct difference in the microbial community between the two systems. The rumen microbiome is known to degrade the xenobiotics faster than other environmental degradation pathways, as revealed through the RDX-degrading ability of the sheep rumen [15].

We did not come across any previous studies in the public domain confirming that the rumen microbes can degrade chlorpyrifos ingested through contaminated feeds, and if it is degraded, then to what extent. To the best of our knowledge, the present study is the first attempt to investigate chlorpyrifos degradation by the rumen microbial consortia and the extent of degradation.

## 2. Materials and Methods

The necessary approval for conducting the studies and collecting rumen fluid was obtained from the Institute Animal Ethics Committee (IAEC) of ICAR-National Institute of Animal Nutrition and Physiology, Bangalore, INDIA, and the Committee for Control and Supervision of Experiments on Animals (CPCSEA), approval no. V-11011(13)/10/2024-CPCSEA-DADF dated 14 October 2024. The ruminal fluid donors were handled in strict adherence to the protocol laid out by the IAEC.

### 2.1. Collection of Rumen Fluid

The donors of ruminal fluids, a source of microbial inoculum for the in vitro studies, were fed on a basal diet consisting of finger millet (*Eleusine coracana*) straw and concentrate mixture in 70:30 (DM basis). Two in vitro studies were conducted in a series to investigate the degradation of chlorpyrifos by the ruminal microbes over a defined incubation period. About 2 L of ruminal fluid containing both solid and liquid fractions was collected 3 h post-feeding from two cannulated Holstein Friesian male cattle (mean BW 613 kg) maintained at the experimental livestock unit of the institute. The rumen fluid was collected in a pre-warmed (39 °C) thermos flask flushed with CO_2_ to maintain anaerobic conditions. The rumen fluid was mixed and filtered through muslin cloth. The filtrate served as the source of ruminal microbial inoculum for the in vitro studies.

### 2.2. Preparation of Stock Solution

A stock solution of 1000 ppm was prepared by dissolving 10 mg of chlorpyrifos (CAS No 285138-81-0, Sigma-Aldrich Production GmbH, Buchs, Switzerland) in 10 mL of LCMS-grade methanol (CAS No 67-56-1, Sigma-Aldrich, St. Louis, MO, USA) in a volumetric flask and sonicated for 1 min. To achieve a concentration of 1 ppm, 0.1 mL stock solution (1000 ppm) was dissolved in 100 mL LCMS-grade methanol, which served as the working standard of chlorpyrifos. The standard linearity curve of 2.5, 5.0, 10.0, 25, 50.0, and 100 ppb was generated by injecting in GC-MS/MS (7000C, Agilent Technologies, Santa Clara, CA, USA). For the experiment, about 14.1 mg of chlorpyrifos was dissolved in 10 mL of LCMS-grade methanol, and the final concentration of 1383.21 ppm was achieved. From the stock solution, the working solutions were prepared for different experiments.

### 2.3. Experiment I

For the experiment, I prepared the working solution by taking 23.61 mL of stock solution and dissolving it in 990 mL of buffered ruminal fluid so that the final concentration of the incubation medium reached 10 ppm. This experiment was conducted without a feed sample, and the ruminal fluid, either with chlorpyrifos or without chlorpyrifos, was incubated for different time periods. A total of 30 glass syringes (100 mL, Heberle, Schwerte, Germany) were incubated in vitro to ascertain the degradation of chlorpyrifos at 0, 24, 48, 72, and 96 h. In addition, 3 syringes containing ruminal fluid without chlorpyrifos, which serve as negative controls, were also prepared for the in vitro incubation. About 30 mL of rumen fluid with or without chlorpyrifos was gently poured into the individual syringe using an automatic bottle top dispenser (Varispenser 50 mL, Eppendorf, Hamburg, Germany). The gas bubbles from the syringes were pushed out, and the position of the piston at the commencement of incubation was recorded. The incubation was set up for 96 h in a Hohenheim-type water bath shaker (Eaga Tools & Instruments, Chennai, India) at 39 °C, and the intermittent shaking was performed every 6 h. Six syringes were removed from the water bath shaker at 0, 24, 48, 72, and 96 h of incubation. To cease the fermentation, these syringes, after removal from the water bath shaker, were immediately placed on the ice. The incubation spent from the individual syringe was filtered through 0.45 μm (Milex-HV, Merck Millipore, Burlington, MA, USA) in a serum vial of 100 mL capacity and placed in the refrigerator at 4 °C until chlorpyrifos analysis.

### 2.4. Experiments II and III

Experiment II was similar to Experiment I, but here, along with the chlorpyrifos-containing buffered ruminal fluid at a similar concentration of 10 ppm, the feed was also incubated to see its impact on chlorpyrifos degradation. The diet for the in vitro studies was prepared by using finger millet straw and concentrate in a 70:30 ratio. About 200 mg of dry feed was weighed and placed in the glass syringes individually; thereafter, 30 mL of chlorpyrifos-containing rumen fluid was added to the syringes. A total of 12 syringes were prepared and incubated in the Hohenheim-type water bath shaker at 39 °C for 24 h. On the termination of incubation, the incubation spent from the individual syringe was filtered through 0.45 μm in a serum vial and placed in the refrigerator at 4 °C until chlorpyrifos analysis.

As methanol was used as a solvent for the spiking of chlorpyrifos in both experiments I and II, and therefore to rule out the influence of methanol on the chlorpyrifos degradation, another experiment (experiment III) was conducted where the chlorpyrifos was directly dissolved in the rumen fluid without using methanol. About 14.1 mg chlorpyrifos was dissolved in 10 mL rumen fluid by sonication for 20 min at a speed of 40 khz (Grant Ultrasonic bath, Cambridge, UK), and a concentration of 1383 ppm was achieved. To achieve the concentration of 5 ppm, about 0.13 mL from the stock solution was dissolved in 360 mL rumen fluid to be used for in vitro incubation. Experiment III was similar to experiment II in all respects except that the methanol was not used for dissolving the chlorpyrifos while preparing the stock solution. A total of 12 syringes containing 200 mg feed along with the 30 mL buffered rumen fluid, which either contained chlorpyrifos or not, were incubated in the Hohenheim-type water bath shaker at 39 °C for 24 h. On the termination of incubation, the spent was filtered and stored at 4 °C until further analysis.

### 2.5. Chlorpyrifos Analysis

Rumen fluid from the donor animals, water used for the preparation of the incubation medium, and feed samples used in the in vitro incubation were analyzed for the presence of chlorpyrifos before conducting the experiments. About 10 mL of each sample was sent for chlorpyrifos analysis at the NABL-accredited laboratory, Eureka Analytical Services, Bengaluru. The chlorpyrifos assay Limit of Detection (LOD) was 0.003 mg/kg, and the Limit of Quantification was 0.01 mg/kg. From each tube, a 5.0 g sample was weighed and transferred into a 50 mL tube. Thereafter, 10.0 mL of Milli-Q water was added and vortexed for five minutes. Subsequently, 10.0 mL acidified acetonitrile was mixed with one packet of QuECHERS (EN15662, Agilent Technologies, Santa Clara, CA, USA) and vortexed for five minutes. The acidified acetonitrile was prepared by taking 0.1 mL acetic acid (Merck, Boston, MA, USA) in 10 mL acetonitrile. The centrifugation was performed at 4200× *g*, 4 °C for five minutes, and the supernatant (4.0 mL) was transferred to the RIA vials (Tarsons, Kolkata, India). The supernatant was preserved at −18 °C for two hours, followed by centrifugation at 4200× *g*, 4 °C for five minutes, and finally 3.0 mL of the supernatant was taken into the RIA vials. About 450 mg MgSO_4_, 75.0 mg PSA, 50.0 mg C18, and 50.0 mg GCB were sequentially added to the RIA vials containing supernatant and vortexed for two minutes. Thereafter, the content was centrifuged for five minutes at 4200× *g*, 4 °C, and one mL of supernatant was transferred to the RIA vials and fully evaporated under N2 gas evaporator (Athena Instruments Pvt., Ltd., Mumbai, India). The residue was reconstituted in one mL of HPLC-grade ethyl acetate (Merck, St. Louis, MO, USA), and 2 μL was injected into the GC-MS/MS (7000C, Agilent Technologies, Santa Clara, CA, USA). The chlorpyrifos was quantified by MRM (multi-reaction monitoring) mode by the MassHunter software version 2.0. The final concentration was auto-calculated considering linearity, dilution factor, and sample weight.

### 2.6. Statistical Analysis

Data on the chlorpyrifos degradation was analyzed using One-way ANOVA in the GraphPad prism, and the mean chlorpyrifos concentration was compared among the different in vitro incubation periods (i.e., 0, 24, 48, 72, and 96 h). The Tukey post-hoc analysis was performed to ascertain the significance among the different time points at a confidence level of 95%. The degradation of chlorpyrifos over 24 h of in vitro incubation in experiment II and III was compared by performing a *t*-test.

### 2.7. Genomic DNA Isolation

About 1.5 mL of the incubation fluid was used for the isolation of genomic DNA using the procedure of Yu and Morrison [16]. In brief, the fluid samples were initially centrifuged at 12,400× *g* for 15 min, and the supernatant was removed. To dissolve the pellet, 1 mL of lysis buffer was added to the tube containing the pellet. This content was transferred to a pre-sterilized screw-cap tube (BioSpec, Bartlesville, OK, USA) containing 0.5 g zirconia beads of 0.1 mm size (BioSpec, Bartlesville, OK, USA). The samples were homogenized using a bead beater (BioSpec, Bartlesville, OK, USA) at the maximum speed for three minutes, followed by incubation at 70 °C for 15 min. Thereafter, the centrifugation was carried out at 12,400× *g,* and the supernatant was transferred in a 2 mL tube. The protein and polysaccharide contaminations were removed by treating with 260 μL of 10 M ammonium acetate and centrifugation at 12,400× *g* for 10 min. The supernatant was removed in another tube, and an equal volume of isopropanol was added, followed by gentle mixing. The precipitated DNA was collected by centrifugation at 4 °C for 10 min at 12,400× *g,* and the pellet was washed with 70% ethanol. Finally, the DNA pellet was dissolved in 100 μL Tris-EDTA buffer, and 2 μL DNase-free RNase (10 mg/mL, Qiagen GmbH, Hilden, Germany) was added for removing the RNA contamination. Subsequently, the QIAamp DNA mini kit (Qiagen GmbH, Hilden, Germany) was used for the isolation of genomic DNA according to the manufacturer’s instructions. The DNA quality was checked with 0.8% agarose gel electrophoresis and quantified with the help of a Qubit 4.0 fluorometer (Invitrogen Corporation, Carlsbad, CA, USA).

### 2.8. Shotgun Sequencing

Metagenome sequencing was performed using NovaSeq 6000 (Illumina Inc., San Diego, CA, USA) at an external facility, i.e., Eurofins Genomics, Bangalore, India. The metagenomic libraries were prepared using the NEBNext^®^ UltraTM II FS DNA Library Prep Kit (New England Biolabs, Ipswich, MA, USA). About 100–500 ng of DNA was fragmented to achieve a size of 350 base pairs. The fragmentation process was carried out by NEBNext Ultra II FS Reaction Buffer (New England Biolabs, Ipswich, MA, USA) and Ultra II FS Enzyme Mix (New England Biolabs, Ipswich, MA, USA) in a PCR. The fragmented DNA was ligated with the NEBNext adapters (New England Biolabs, Ipswich, MA, USA) by combining 35 μL of fragmented DNA with NEBNext Ultra II Ligation Master Mix (New England Biolabs, Ipswich, MA, USA). The mixture was then incubated at 20 °C for 15 min, followed by ligation with adaptors. The libraries were PCR amplified with the index primers (i5 and i7) by using the following conditions: initial denaturation at 98 °C, 30 s; denaturation at 98 °C, 10 s; annealing at 65 °C, 75 s; and final extension at 65 °C, 5 min. The libraries were evaluated on the Agilent 4150 Tape Station, pooled, and sequenced on NovaSeq 6000 (Illumina, San Diego, CA, USA) using 2 × 150 bp chemistry.

### 2.9. Bioinformatic Analysis

The Illumina paired-end metagenome sequencing data containing leftover adapters, low-quality bases, Ns, and polyG stretches in the reads with short inserts were cleaned using fastp v0.23.4 [17] using command line options ‘-e 30, -l 70 -g -3 -W 4 -M 20 -h’ for average read quality score, minimum length filter, removing polyG stretches, sliding window for average quality, cut mean quality, and html report generation, respectively. For removing the host contamination from the reads, the quality-filtered data were mapped against the reference created using cattle genome assembly ARS-UCD1.2 (RefSeq assembly accession: GCF_002263795.1) in Hisat2 v2.2.1 [18]. The unmapped paired-end and single-end reads were saved for further analysis using the output option ‘--un-conc-gz’. The clean reads obtained from the Hisat2 output were taxonomically classified using the Kraken2 database at BV-BRC v3.55.7 [19] following the K-mer-based approach, and the taxonomic affiliation was assigned by the lowest common ancestor method [20]. The Kraken output files were resolved to different taxonomic ranks of bacterial and archaeal domains in Pavian v1.0 [21], and the contingency tables at the phylum, order, and genus levels were prepared for downstream analysis and visualization. The contingency tables were filtered for the counts less than 4 with a minimum prevalence of the taxa in at least 20% of the samples and a low variance filter set at 10%. The filtered data were centered log-ratio transformed in MicrobiomeAnalyst v2.0 [22]. For each taxonomic level, the samples were merged into groups, and the composition bar plots were constructed by scaling the bars. Further, the abundance at different taxonomic ranks between the groups was compared for statistically different changes after applying the intrinsic normalization method of DESeq2 in MicrobiomeAnalyst v2.0. The significance cutoff was set at a false discovery rate of < 0.05. The alpha diversity was estimated at the genus level by the Shannon index, and the sample dissimilarity between the groups was estimated by beta diversity based on the Bray–Curtis dissimilarity matrix.

The alpha diversity was represented as pooled box plots, and the beta diversity was graphically represented in PCoA ordination. The statistical comparison between the alpha diversity and beta diversity was performed by the Kruskal–Wallis test and the PERMANOVA, respectively. To find the difference in the key microbial biomarkers between the groups, the linear discriminant analysis (LEfSe) was performed in MicrobiomeAnalyst v2.0 and then linear discriminant analysis for effect size normalization with an LDA score > 2.0 [23].

### 2.10. Metagenome Gene Annotation

The microbial genes in the metagenome data were predicted and assigned functional roles using MetaErg v1.2.3 [24]. Briefly, the clean read files, free of host contamination, were assembled into contigs using MEGAHIT (v1.2.9, [25]. The contigs below >300 bp in length were filtered out. The clean reads were mapped against the assembled contig sequences using HISAT2 (v2.2.2, [18]. The BAM files were used for generating depth files using BBmap v35.85 [26] ‘pileup.sh’ command. The functional annotation of the samples was performed in MetaErg—the metagenome annotation pipeline. The assigned KEGG Orthology (KO), Enzyme Commission (EC), and Gene Ontology (GO) and their NCBI taxonomy information relevant to chlorpyrifos metabolism/degradation were extracted, and the contribution of different phyla and orders to the detected KO, EC, and GO categories was presented in stacked bar charts, and the category-wise gene abundance was depicted in the corresponding bar plots.

## 3. Results

The chlorpyrifos content in the ruminal fluid, water, and feed samples used for the in vitro experiments was screened by GC MS/MS. The chlorpyrifos concentration in the above samples remained below the level of quantification (BLQ, i.e., 0.01 mg/kg), indicating that none of the inputs were contaminated with chlorpyrifos.

### 3.1. Chlorpyrifos Degradation

**Figure 1:**
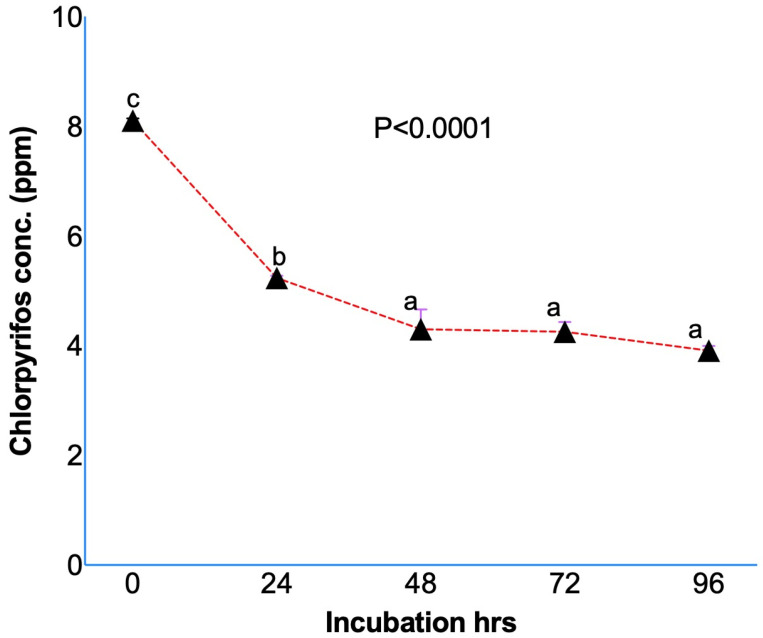
Chlorpyrifos degradation during in vitro incubation over 24 h by dissolving chlorpyrifos in methanol and incubating along with buffered rumen fluid without any feed sample. Chlorpyrifos concentrations bearing different superscripts a, b, c at different incubation hours represent the significant difference among the incubation hours.

**Figure 2:**
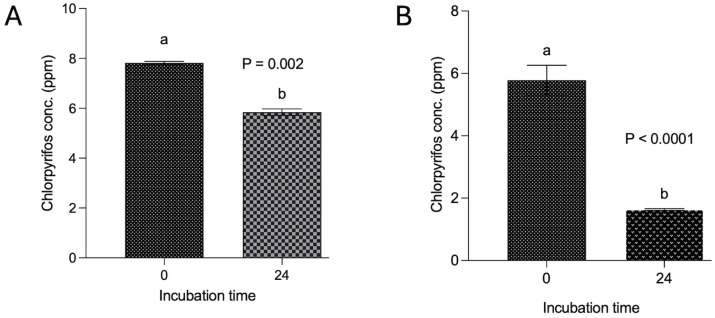
Degradation of chlorpyrifos with and without methanol spiking over 24 h. Incubation in panel (**A**) was conducted by dissolving chlorpyrifos in methanol and then in vitro incubation along with buffered rumen fluid and 200 mg feed over 24 h, whereas in panel (**B**), the incubation was achieved by dissolving chlorpyrifos in ruminal fluid without using methanol and then incubation along with buffered rumen fluid and a 200 mg feed sample. Chlorpyrifos concentrations bearing different superscripts a, b at different incubation hours represent the significant difference between the incubation hours.

Results from the in vitro studies confirmed the degradation of chlorpyrifos while initially dissolved in methanol (Figure 1) and ruminal fluid without (Figure 2A) or with (Figure 2B) a feed sample. The initial concentration of the chlorpyrifos in the buffered rumen fluid at 0 h was 8.10 ± 0.039 ppm, which after 24 h of in vitro incubation decreased to 5.23 ± 0.042 ppm (Figure 1). The chlorpyrifos concentration was recorded as 4.29 ± 0.364, 4.25 ± 0.177, and 3.91 ± 0.082 ppm at 48, 72, and 96 h of incubation, respectively. The results from the experiment I demonstrated a significant (*p* < 0.0001) degradation of the chlorpyrifos over 24 h of in vitro incubation with the rumen fluid. About a 35.4% decrease in the chlorpyrifos concentration as compared to the initial concentration was recorded at 24 h of incubation. Similarly, the chlorpyrifos concentration at 48 h was further decreased by 47.0% as compared to the initial concentration at 0 h. The chlorpyrifos concentration (ppm) at 48 h of incubation was significantly (*p* < 0.0001) lower than the concentration at 0 and 24 h of incubation. However, the chlorpyrifos concentration at 72 and 96 h was not significantly decreased as compared to the concentration at 48 h.

Since the experiment I was conducted without feed to get an idea if the chlorpyrifos can be degraded in the ruminal fluid, which possesses anaerobic microbiota, the investigation was further extended to additional experiments with feed and chlorpyrifos either dissolved in methanol or ruminal fluid.

The findings from experiment II revealed a degradation of 25.4% of chlorpyrifos over the 24 h in vitro incubation with feed and buffered ruminal fluid (Figure 2A). The decrease in the chlorpyrifos concentration (ppm) at 24 h incubation was significantly (*p* < 0.002) lower than the initial concentration. However, the results from another experiment where chlorpyrifos was dissolved in the ruminal fluid itself without using methanol demonstrated a substantial decrease (*p* < 0.0001) in the chlorpyrifos concentration from the initial 5.78 ppm to 1.64 ppm over 24 h incubation (Figure 2B). A significant (*p* < 0.0001) degradation of 71.6% was achieved by dissolving the chlorpyrifos in ruminal fluid and incubating with feed and ruminal fluid over 24 h.

### 3.2. Rumen Metagenome

In the present study, a total of 528 million sequencing reads of data were generated with an average of 44.1 million raw reads per sample (Supplementary Table S1). After quality filtration, on average 5.55% of reads per sample were dropped due to poor quality, short length, and adapter contamination. Further, 0.01% of the metagenomic reads per sample were dropped due to the host contamination removal. Subsequently, about 94.4% of the high-quality paired and single-end metagenomic reads were subjected to retrieve the information on compositional abundances.

**Figure 3:**
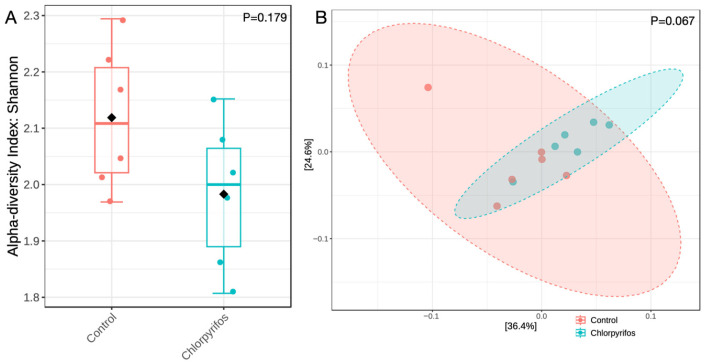
Panel (**A**) represents the alpha diversity index of the rumen microbiota assessed through Shannon index method, whereas Panel (**B**) represents the beta diversity using the Bray-Curtis dissimilarity index. In control treatment, the in vitro incubation was set up using feed and buffered rumen fluid without chlorpyrifos, whereas another treatment consisted of feed, buffered rumen fluid, and chlorpyrifos for the in vitro incubation over 24 h. *p*-value in the panels indicates whether the alpha or beta diversity was different between the two groups.

The similar alpha diversity index between the control and chlorpyrifos treatments revealed that the richness and the evenness of the microbial population were not affected (*p* = 0.179) by the contamination of rumen fluid with chlorpyrifos pesticide at a concentration of 5.78 ppm (Figure 3A). Similarly, beta diversity (Figure 3B) analyzed the differences in microbial composition between two treatments, and the non-significant *p*-value of 0.067 indicated that the microbiota composition was also not different in this study.

**Figure 4:**
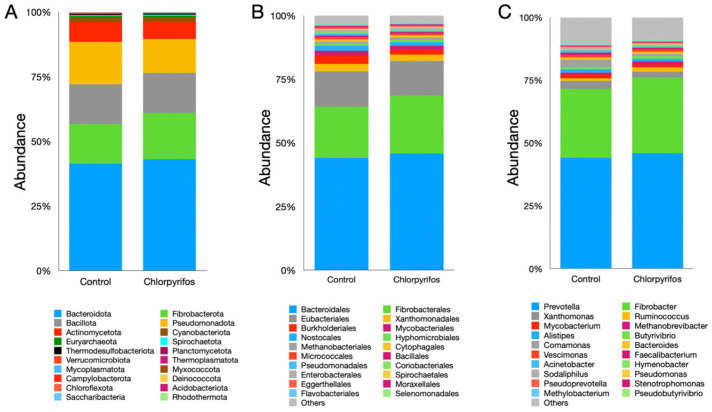
Effect of contamination of ruminal fluid with chlorpyrifos on the compositional abundances of rumen microbiota. Panel (**A**) represents the microbial abundances at phylum level, panel (**B**) at the order, and panel (**C**) at the genus level. In control treatment, the in vitro incubation was set up using feed and buffered rumen fluid without chlorpyrifos, whereas another treatment consisted of feed, buffered rumen fluid, and chlorpyrifos for the in vitro incubation over 24 h.

In this study, a total of 20 microbial phyla were identified (Supplementary Table S1), where Bacteroidota, Fibrobacterota, Bacillota, and Pseudomonadota constituted the largest fraction of the microbiota (Figure 4A). The Bacteroidota phylum alone represented more than 40% of the total microbiota; however, their abundance was similar (pFDR = 0.0711) between the two treatments. Similarly, the abundances of Fibrobacterota (pFDR = 0.0711), Bacillota (pFDR = 0.101), and Pseudomonadota (pFDR = 0.563) phyla also did not differ significantly between the treatments. The chlorpyrifos contamination in this study also did not exhibit any significant (pFDR = 0.0711) changes in the abundance of archaeal phylum Euryarchaeota. Likewise, the abundances of other microbial phyla were also found to be similar between the treatments (Figure 4A, Supplementary Table S1).

A total of 57 microbial orders were identified in the present study (Figure 4B, Supplementary Table S1). Among the orders, the Bacteroidales represented the largest fraction; however, the chlorpyrifos contamination did not affect (pFDR = 0.120) their abundance between treatments. Similarly, the abundances of other prominent microbial orders such as Fibrobacterales (pFDR = 0.381), Eubacteriales (pFDR = 0.464), and Xanthomonadales (pFDR = 0.941) were also similar between the treatments and not affected by the chlorpyrifos contamination of the ruminal fluid. Likewise, the abundance of the prominent archaeal order Methanobacteriales was also similar (pFDR = 0.120) between the control and chlorpyrifos treatments. Overall, no significant difference in the microbiota at the order level was reported in the present study.

A metagenomic study identified a total of 120 microbial genera in the ruminal fluid. Among the genera, *Prevotella* was the single largest genus, which constituted 44–45% of the total ruminal microbiota (Figure 4C, Supplementary Table S1). The contamination of ruminal fluid with chlorpyrifos did not affect (pFDR = 0.120) the abundance of the *Prevotella* genus in the present study. The *Fibrobacter* (pFDR = 0.126) and *Xanthomonas* (pFDR = 0.162) were the next abundant microbial genera reported with a similar abundance between the two treatments. At the genus level, the abundance of *Ruminococcus* was significantly different (pFDR = 0.007) between the control and chlorpyrifos treatments (Figure 4C, Supplementary Table S1). Among the archaeal genera, *Methanobrevibacter* was the most prominent genus, which constituted 0.77–1.14% of the total microbiota; however, there was no significant difference (pFDR = 0.468) between the two treatments in their abundance. Similarly, the abundances of other genera also did not differ between the two treatments (Supplementary Table S1).

### 3.3. Gene Annotation

**Figure 5:**
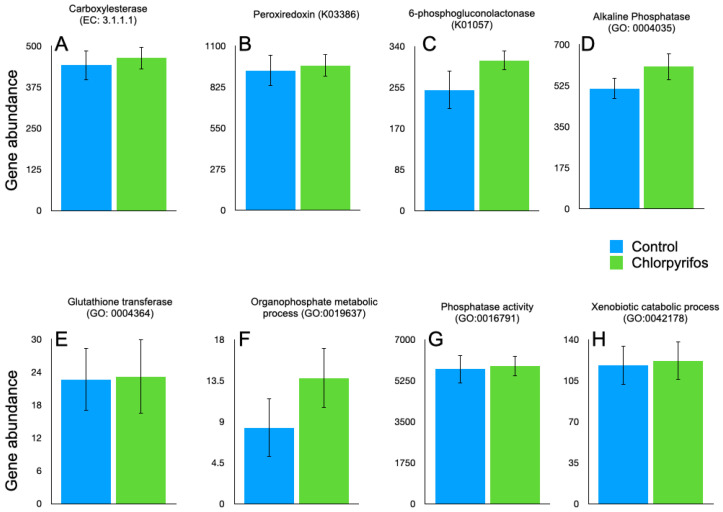
Abundance of the EC, GO, and KOs associated with chlorpyrifos degradation in the rumen metagenome data. The abundances are calculated from the metagenomic contig coverage values estimated by mapping the sequencing data against the contigs, and the predicted genes from the contigs were mapped against the EC, KO, and GO databases. (**A**): abundance of carboxylesterase (EC 3.1.1.1); (**B**): peroxiredoxin (K03386); (**C**): 6-phosphogluconolactonase (K01057); (**D**): alkaline phosphatase (GO:0004035); (**E**): glutathione transferase (GO:0004364); (**F**): organophosphate metabolic process (GO:0019637); (**G**): phosphatase activity (GO:0016791); (**H**): xenobiotic catabolic process (GO:0042178).

**Figure 6:**
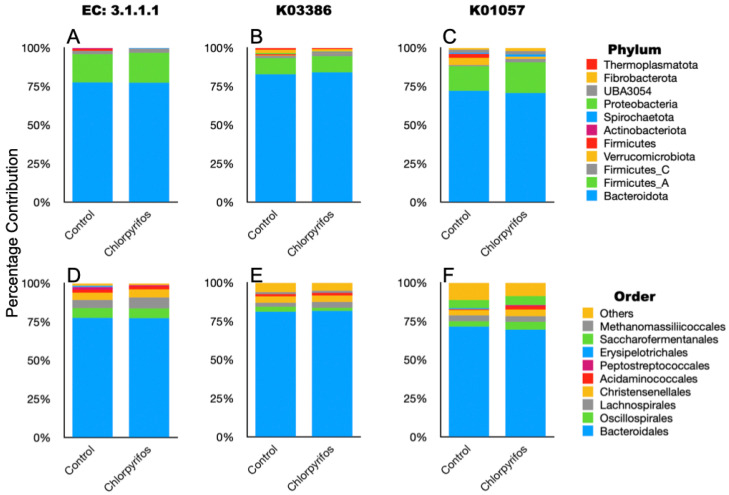
Taxonomic composition of the ruminal microbes that contributed to the selected EC and KOs associated with chlorpyrifos degradation in the rumen metagenome data. (**A**): phylum-level composition of metagenome members contributed to EC 3.1.1.1; (**B**): phylum-level composition of metagenome members contributed to K03386; (**C**): phylum-level composition of metagenome members contributed to K01057; (**D**): order-level composition of metagenome members contributed to EC 3.1.1.1; (**E**): order-level composition of metagenome members contributed to K03386; (**F**): order-level composition of metagenome members contributed to K01057.

In annotated metagenome data, we have tried to identify the genes involved in the enzymatic degradation of chlorpyrifos with their EC numbers. The search was made for the previously reported EC 3.1.8.1 (organophosphorus hydrolase), EC 3.1.3.1 (phosphatase), EC 1.14.13.- (monooxygenases), EC 1.1.1.- (dehydrogenases), and EC 3.1.1.- (esterases). However, the genes affiliated with EC 3.1.8.1, EC 3.1.3.1, EC 1.14.13.-, and EC 1.1.1.- were not present in our metagenome data. In the present study, only carboxylesterase (EC 3.1.1.1) was present in the metagenome data (Figure 5). The gene abundances for EC 3.1.1.1 were 442 ± 44.1 and 464 ± 34.4, respectively, in the control and chlorpyrifos treatments. The gene abundances for EC 3.1.1.1 were numerically higher in the chlorpyrifos treatment but not significantly (*p* = 0.693) different from the control (Supplementary Table S1). The genes for carboxylesterase in the metagenome were contributed by the microbes affiliated with the two major phyla Bacteroidota and Firmicutes (Figure 6, Supplementary Table S1). At the order level, Bacteroidales, Oscillopirales, Lachnospirales, and Christensenellales contributed to the genes encoding carboxylesterase (Figure 6, Supplementary Table S1).

Based on the reported KEGG orthologs (KO) involved in the chlorpyrifos degradation, we searched the metagenome gene catalogue for a total of seven KO (K07099, K01077, K00540, K01093, K00128, K03386, and K01057) in the annotated metagenome data. The K07099, K01077, K00540, K01093, and K00128 that encode for organophosphorus hydrolase, phosphatase, monooxygenases, esterases, and dehydrogenases were not identified in this study. However, K03386 and K01057, encoding peroxiredoxin and 6-phosphogluconolactonase, respectively, were detected in the metagenome (Figure 5). The Bacteroidota, Firmicutes, Fibrobacterota, and Thermoplasmatota phyla contributed to the K03386, whereas the K01057 was mostly associated with the Bacteroidota, Firmicutes, Verrucomicrobiota, Fibrobacterota, and UBA3054 phyla (Figure 6, Supplementary Table S1).

In the metagenome, the gene ontology (GO) for the chlorpyrifos degradation, such as GO:0046434, GO:0006805, GO:0009636, GO:0016787, GO:0003824, GO:0004035, GO:0004364, GO:0019637, GO:0016791, and GO:0042178, was identified. The GO:0046434, GO:0006805, GO:0009636, GO:0016787, and GO:0003824 were absent in the metagenome. On the contrary, GO:0004035, GO:0004364, GO:0019637, GO:0016791, and GO:0042178 were present in the metagenome (Figure 5). The GO present in the metagenome encodes for the enzymes alkaline phosphatase, glutathione transferase, organophosphate metabolic process, phosphatase activity, and xenobiotic catabolic process, respectively. However, the abundances of these GO were similar between the treatments (Supplementary Table S1).

## 4. Discussion

Chlorpyrifos, a broad-spectrum organophosphorus pesticide, due to its wide use, is considered as one of the major feed and environmental contaminants [27]. The persistence of chlorpyrifos in soil is reported to be moderate to high [28] depending upon the soil type, soil pH, microbiota, temperature, and moisture [29], and due to low water solubility and a strong tendency for binding with organic matter, it can persist for quite a long time [30]. The pesticide is transferred from soil to the plants through root uptake, direct adsorption on plant surfaces, and contamination during application. Accumulation of chlorpyrifos in feed and fodder poses serious health implications, particularly to livestock. The contaminating pesticide residues in animal feed could be a source of pesticide residues in milk [31,32]. Although chlorpyrifos degradation is extensively studied in the soil [7,8,33], studies investigating the chlorpyrifos degradation in the rumen and its mechanism are still scanty.

The rumen is strictly an anaerobic compartment harboring diverse microbiota, including bacteria, protozoa, fungi, archaea, and bacteriophages. These microbes act syntrophically in the rumen ecosystem and perform important functions such as feed fermentation, volatile fatty acid production, microbial protein synthesis, and removal of fermentative gases [34,35]. In addition, the rumen microbial consortia are also reported to have metabolic capabilities for degrading the xenobiotic compounds, including pesticides, and the ability can be acquired by the ruminal microflora through continuous exposure [36] and can be transferred by transfaunation [37,38]. The ruminal environment can be ideal for anaerobic degradation of chlorpyrifos, as the degradation is effective over a narrow range (5.5–8.5) of pH [39] and temperature between 30 and 37 °C [40,41]. The degradation of chlorpyrifos up to a significant extent (71%) over 24 h incubation was demonstrated in the present study. This rate of degradation is faster than the anaerobic degradation of chlorpyrifos in soils [42]. Further, the degradation of chlorpyrifos in the rumen fluid inoculum in experiment I was 64% as compared to 71% in experiment II, where additional feed material was incubated with the chlorpyrifos-spiked buffered rumen fluid. This result is substantiated by in-soil degradation of chlorpyrifos; the degradation is faster in the presence of other nutrients that support faster growth of microbes [39,43]. While comparison of chlorpyrifos degradation in experiment II, wherein chlorpyrifos was solubilized in methanol versus directly in rumen fluid, revealed that the degradation over a 24 h period was less in incubation where methanol was used as a solvent (25.4) compared to the other incubation without using methanol as a solvent (71.6%). The presence of hydrocarbons/alcohols reduced the chlorpyrifos degradation [44]. Results of this study clearly established that the rumen microbes can degrade chlorpyrifos through the microbial enzymes. The degradation of chlorpyrifos due to the poor water solubility is not possible, as it is almost insoluble in water [45]. The microbial degradation of chlorpyrifos is in consonance with Megharaj et al. [46], who reported that the heat sterilization of rumen fluid lost the hydrolytic and degradation activity. Hydrolysis is the predominant mechanism of chlorpyrifos degradation [47]. The hydrolytic degradation of chlorpyrifos is majorly performed by the organophosphorus hydrolases through a nucleophilic strike on the phosphorus atom [48,49]. However, there is no report in the public domain confirming the occurrence of organophosphorus hydrolases and degradation of chlorpyrifos in the rumen. In addition, certain bacteria and fungi also have capabilities to perform the oxidative desulfuration of chlorpyrifos [50]. However, the anaerobic conditions of the rumen restrict the oxidative transformations, and therefore, the reductive dichlorination appears to be the complementary pathway for chlorpyrifos degradation in the anaerobic environment [51]. Further, the dichlorination is faster in anaerobic conditions [52]. The sulfate-reducing bacteria in the rumen [53] may also contribute to the reductive degradation of chlorpyrifos [54].

*Pseudomonas* have been reported as the most efficient degraders of chlorpyrifos through their organophosphorus hydrolases enzyme [55]. Further, *Enterobacter* [10] and *Bacillus* [56] are also potential degraders of the chlorpyrifos. The detection of the Pseudomonadota phylum in our metagenome confirmed that the chlorpyrifos-degrading *Pseudomonas* and *Enterobacter* were present in the rumen. Similarly, *Bacillus* affiliated with the phylum Bacillota (Firmicutes) was also detected in the rumen metagenome. The presence of chlorpyrifos-degrading microbial phylum indicated that the rumen possesses microbes capable of degrading chlorpyrifos pesticide. The absence of EC 3.1.8.1, EC 3.1.3.1, EC 1.14.13.-, and EC 1.1.1.- in the metagenome revealed that the organophosphorus hydrolase, phosphatase, monooxygenase, and dehydrogenase enzymes were not involved in the degradation of chlorpyrifos, and only EC 3.1.1.1 carried out the degradation of chlorpyrifos in the present study, as only the carboxylesterase was detected in the present study. Singh [57] reported that the carboxylesterases are important in degrading the xenobiotic, including organophosphorus pesticides. Carboxylesterases are reported to hydrolyze the phosphotriester bond in chlorpyrifos molecules and produce less toxic alcohols and acids [58,59]. The substantiative evidence of chlorpyrifos degradation by carboxylesterase was established by Wang et al. [60]; the authors have shown that the immobilized carboxylesterases degraded 90% of chlorpyrifos content in the farmland water. Further, the presence of GO:0004035, GO:0004364, GO:0019637, GO:0016791, and GO:0042178 in the metagenome strengthens that the chlorpyrifos degradation in the present study was primarily assigned to the rumen microbiota. These GO categories are related to the enzymes alkaline phosphatase, glutathione transferase, organophosphate metabolic process, phosphatase activity, and xenobiotic catabolic process, which is associated with the chlorpyrifos degradation.

Out of the seven KOs, two (K03386 and K01057) were detected in the annotated rumen metagenome. Therefore, the KO for organophosphorus hydrolase, phosphatase, monooxygenase, esterases, and dehydrogenases remains undetected in the metagenome. The two Kos, i.e., K03386 and K01057, detected in the metagenome encode for peroxiredoxin and 6-phosphogluconolactonase, respectively. During the bacterial degradation of chlorpyrifos, the enzyme peroxiredoxin helps in neutralizing the reactive oxygen species [61] so that the detoxification process is continued. In the metagenome, the major contributors to the peroxiredoxin were Bacteroidota, Firmicutes, Fibrobacterota, and Thermoplasmatota phyla, whereas Bacteroidota, Firmicutes, Verrucomicrobiota, Fibrobacterota, and UBA3054 phyla contributed to the K01057.

This in vitro study provides insights into the rumen microbiota involved in the chlorpyrifos degradation and the initial clue that the rumen microbes are capable of degrading chlorpyrifos. However, studies with the pure microbial isolates of rumen origin affiliated with the phyla Pseudomonadota and Bacillota are warranted to determine the efficiency of different microbes for chlorpyrifos degradation. Apart from the bacteria and archaea, the ruminal microbiome also contains protozoa and anaerobic fungi. We did not come across any report on protozoal degradation of chlorpyrifos. Although studies in soil and other environments did report aerobic fungi in the degradation of chlorpyrifos and TCP. The rumen contains only anaerobic fungi belonging to the phylum Neocallimastigomycota, and the fungi are limited to only 6 genera, namely, *Neocallimastix*, *Piromyces*, *Orpinomyces*, *Caecomyces*, *Cyllamyces*, and *Anaeromyces*. No report is available on the chlorpyrifos degradation by the ruminal fungi. Further, the animal studies in different species with the variable levels of chlorpyrifos are also warranted to confirm the efficacy of rumen microbes in mixed syntrophy and also determine the threshold capabilities of the ruminal microbes.

## 5. Conclusions

From the in vitro study, it can be inferred that the chlorpyrifos pesticide can be degraded in the rumen to a significant extent. Rumen microbiota affiliated with Pseudomonadota and Bacillota are actively involved in the ruminal degradation of chlorpyrifos via carboxylesterase (EC 3.1.1.1). Since the concentration of the chlorpyrifos used in the study did not influence the microbial diversity or composition between the control and chlorpyrifos-spiked samples, it indicates the innate ability of the ruminal microbiome to degrade the chlorpyrifos. This study was restricted to an in vitro setup; hence, the animal studies in different species with the variable levels of chlorpyrifos are also warranted to confirm the efficacy of rumen microbes in mixed syntrophy and also determine the threshold capabilities of the ruminal microbes. The metagenomic approach can only reveal the presence of degrading genes; however, their actual expression needs to be evaluated through the metatranscriptomic approach. The metabolites of the chlorpyrifos degradation were not estimated in the present study, which limits the identification of the actual chlorpyrifos degradation mechanism in rumen. Tracing the chlorpyrifos degradation metabolites in different organs and milk would be helpful in establishing the ability of ruminants to offset the chlorpyrifos or its residue contamination in the animal-origin products.

## Data Availability

The datasets presented in this study can be found in online repositories with the accession number PRJNA1393601. The metagenome data with accession number(s) is available in the repository/repositories and can be found at: https://www.ncbi.nlm.nih.gov/sra/PRJNA1393601, accessed on 25 December 2025.

## Supplementary Materials

The following supporting information can be downloaded at: https://www.mdpi.com/article/10.3390/microorganisms14030581/s1, Table S1: Read stats, taxonomic affiliation, and functional analysis data of the microbiota.

## Abbreviations

The following abbreviations are used in this manuscript:

KEGG	Kyoto Encyclopedia of Genes and Genomes
KO	KEGG Orthologs
GO	Gene Ontology
EC	Enzyme Commission (numerical classification of enzymes based on chemical reactions they catalyze)
QuECHERS	Quick, Easy, Cheap, Effective, Rugged, and Safe, sample preparation for GC-MS
ppm	Parts per million (mg/kg)
pFDR	False Discovery Rate corrected *p* value
